# An Authentication Protocol for Future Sensor Networks

**DOI:** 10.3390/s17050979

**Published:** 2017-04-28

**Authors:** Muhammad Bilal, Shin-Gak Kang

**Affiliations:** Electronics and Telecommunications Research Institute, University of Science and Technology, 218, Gajeong-ro, yuseong-gu, Daejeon 34129, Korea; sgkang@etri.re.kr

**Keywords:** authentication, sensor networks, network security, key distribution, privacy, BAN logic

## Abstract

Authentication is one of the essential security services in Wireless Sensor Networks (WSNs) for ensuring secure data sessions. Sensor node authentication ensures the confidentiality and validity of data collected by the sensor node, whereas user authentication guarantees that only legitimate users can access the sensor data. In a mobile WSN, sensor and user nodes move across the network and exchange data with multiple nodes, thus experiencing the authentication process multiple times. The integration of WSNs with Internet of Things (IoT) brings forth a new kind of WSN architecture along with stricter security requirements; for instance, a sensor node or a user node may need to establish multiple concurrent secure data sessions. With concurrent data sessions, the frequency of the re-authentication process increases in proportion to the number of concurrent connections. Moreover, to establish multiple data sessions, it is essential that a protocol participant have the capability of running multiple instances of the protocol run, which makes the security issue even more challenging. The currently available authentication protocols were designed for the autonomous WSN and do not account for the above requirements. Hence, ensuring a lightweight and efficient authentication protocol has become more crucial. In this paper, we present a novel, lightweight and efficient key exchange and authentication protocol suite called the Secure Mobile Sensor Network (SMSN) Authentication Protocol. In the SMSN a mobile node goes through an initial authentication procedure and receives a re-authentication ticket from the base station. Later a mobile node can use this re-authentication ticket when establishing multiple data exchange sessions and/or when moving across the network. This scheme reduces the communication and computational complexity of the authentication process. We proved the strength of our protocol with rigorous security analysis (including formal analysis using the BAN-logic) and simulated the SMSN and previously proposed schemes in an automated protocol verifier tool. Finally, we compared the computational complexity and communication cost against well-known authentication protocols.

## 1. Introduction

**Figure 1:**
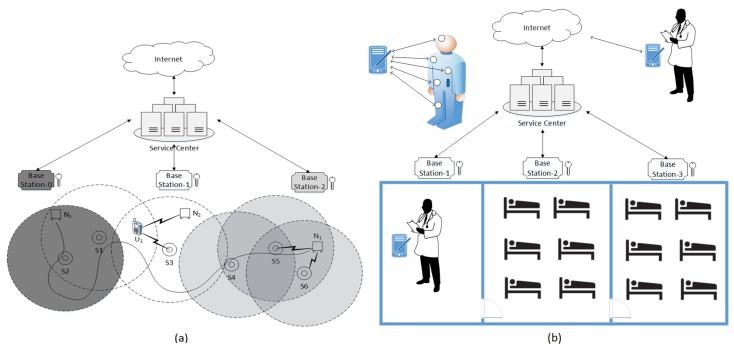
(**a**) An example scenario of a Wireless Sensor Network (WSN). Sensor node $N_{1}$ is initially authenticated by $BS_{1}$; while moving across the network it is re-authenticated by the re-authentication ticket. At the final destination $N_{1}$ shares data with multiple sink nodes while user node $U_{1}$ collects data from sensor node $N_{2}$ and sink node $S_{3}$. (**b**) An application scenario of Secure Mobile Sensor Network (SMSN) in a smart hospital. Authorised doctors and nursing staff can access the data from sensor nodes using a user device.

Wireless sensor networks (WSNs) consist of a vast number of distributed sensor nodes. Each sensor node is an autonomous system that monitors and collects data from the surrounding environment. In wireless sensor networks, the sensor nodes have limited computational power and communication capabilities; hence, most of the conventional cryptographic mechanisms and security protocols are not suitable for resource limited WSNs. For instance, very efficient public key algorithms, such as ECC [1], need a fraction of a second to execute the encryption/decryption procedures, while a symmetric key algorithm, such as RC5 [2], needs only a fraction of a millisecond to perform encryption and decryption procedures [3,4,5]. In a sensor network where devices have limited resources, the asymmetric cryptographic functions must be used wisely; for instance, use the asymmetric cryptography when an authentication responder already verified the initiator and wants to share secret information. If the responder does not authenticate the initiator and the initiator uses the asymmetric cryptography to exchange a message, the network becomes vulnerable to DOS attacks [5,6]. The WSN related contraints mentioned above are known to the research community. The currently available authentication protocols are designed for the autonomous WSN from the perspective of the above constraints. Moreover, in the near future in the realization of the vision of emerging technologies such as IoT, D2D, smart home and smart cities, WSNs will provide an invaluable service by acting as a virtual layer between the physical world and the computational devices [7,8,9]. However, integration of WSNs with IoT will bring forth a new kind of WSN architecture and stricter security requirements; for instance, in a smart hospital (as shown in Figure 1b) a sensor node or a user node may require the establishment of multiple concurrent secure data sessions. To establish a secure data session, authentication is the first step. In a dynamic, mobile WSN environment, where sensors and user nodes can establish multiple concurrent connections, a node moving across the network undergoes the authentication check multiple times and the frequency of the re-authentication process increases in proportion to the number of concurrent connections. Moreover, to establish multiple data sessions, it is essential that a protocol participant has the capability of running multiple instances of the protocol run, which makes the security issue even more challenging. Thus, it is essential to adopt a secure yet lightweight authentication procedure that especially reduces the computational time and communication at the mobile sensor node. The currently available authentication protocols were designed for the autonomous WSN and do not account for these new emerging challenges. This work presents a novel authentication protocol suite called the Secure Mobile Sensor Network (SMSN) Authentication Protocol. The SMSN protocol suite consists of six protocols: three protocols deal with mobile sensor node authentication with sink nodes and the other three deal with user node activation and authentication with the base station, sink nodes, and sensor nodes. In the SMSN, mobile sensors and user nodes can join and leave the system dynamically and can establish secure multiple concurrent connections. After the initial authentication, a mobile sensor or a user node can move across the network and get re-authenticated by a simple ticket-based re-authentication protocol; for instance, a user node can establish concurrent connections with multiple sink and sensor nodes using a re-authentication ticket issued during the initial-authentication protocol run. To establish multiple connections, a node is allowed to run multiple instances of the protocol; consequently, we introduce extra design requirements to meet the goals of a secure authentication protocol. In this paper, we also present an efficient and lightweight key generation and distribution mechanism. In the key generation protocol, a commitment key is generated by a group of participants (the base station and sink nodes) using an irreversible function; the key agreement and key retrieval protocol are the same as that employed in [10]. The commitment key is further applied to drive multiple time-based encryption keys, for example, the ticket encryption key and session key between the sink and user/sensor are derived from the commitment key. The time dimension in the protocol increases the security of the protocol; although the group members do not need to be tightly or loosely time synchronized. To determine the security of the protocol in this study, we performed a rigorous security analysis and also simulated the SMSN and previously proposed schemes [11,12,13,14,15,16] in an automated security protocol analysis tool called Scyther [17,18], which is a powerful state-of-art tool that finds attacks for defined protocol properties. We observed that our authentication protocol is secure, and it achieves all the objectives of an authentication protocol, which are defined as protocol claims in Section 5.3; for a detailed description of protocol claims, please refer to [19,20]. We also compared the efficiency of the SMSN in terms of computational time and communication complexities as discussed in [11,12,13,14,15,16]. The remainder of this paper is organized as follows. Section 2 presents the related work. In Section 3, we present a brief system overview and problem statement. Section 4 describes the proposed scheme with a detailed discussion. In Section 5, we assess the strength of our scheme against the known attacks. Section 6 presents an efficiency analysis that compares a few interesting schemes with our scheme. Finally, we provide concluding remarks in Section 7.

## 2. Related Work

Typically, WSNs are comprised of distributed devices with limited resources. Most of the conventional cryptographic mechanisms and security protocols are computationally expensive and are not suitable for resource-limited WSNs. In the recent past, the research community proposed several authentication protocols [11,12,13,14,15,16,21,22,23,24,25,26,27] that provide security in a WSN environment. Since the sensor nodes have low computational time, storage and communication capabilities, it is essential to design an efficient and lightweight yet secure authentication mechanism. From the point of view of computational and communication complexity, the authentication procedure in a wireless network with a mobile sensor and user node is an expensive task. A node moving across the network undergoes multiple authentication checks. Thus, it is essential to adopt a secure yet lightweight authentication procedure that especially reduces the computational and communication resources at the mobile sensor node. In [28] the authors discussed various anomaly detection techniques for flat and hierarchical wireless sensor networks, but detection techniques are not sufficient for several security threats. However, for a secure system together with detection methods, prevention techniques such as authentication is also vital; various authentication protocols for WSNs were proposed in [11,12,13,14,15,16,21,22,23,24,25,26,27]. In 2006, Wong [21] proposed a user authentication scheme for a dynamic WSN. The scheme is password based and employs lightweight cryptographic hash and XOR operations. Later on, Tesng et al. [11] and Das [22] identified that the Wong [21] scheme had various weaknesses and was vulnerable to replay attacks and forgery attacks, and the user password is known to the sensor node and can be revealed by any sensor node. Tesng et al. [11] proposed an improved version to mitigate the weaknesses that posed security threats in the Wong [21] scheme. The scheme of Tesng et al. [11] is also a password-based scheme, but the password is not revealed to the sensor nodes, and they also introduced a new phase of password change. Nevertheless, the scheme of Tesng et al. [11] is weak against replay attacks, impersonation attacks and forgery attacks [29]. Moreover, the scheme does not provide a mutual authentication between the gateway (GW) and sensor node (SN). In 2009, Das [22] proposed a two-factor user authentication scheme in which legitimate users can register and log in to the remotely deployed sensor nodes to access the collected data. From the point of view of computational and communication complexity, the scheme is reasonably efficient. The author claimed the scheme was secure against various kinds of attacks. However, later work [30,31,32] suggested that the scheme was vulnerable to different types of attacks, including impersonation, password guessing, insider, and parallel session attacks. Moreover, it did not provide the mutual authentication between the GW and sensor nodes. In 2010, Yoo et al. [12] proposed a user authentication scheme for WSNs and analyzed the protocol using BAN logic [33]. However, the BAN logic provided a foundation for the formal analysis of security protocols, but in the case of authentication, various attacks could slip through the BAN logic [34,35]. The scheme of Yoo et al. provided mutual authentication between the GW and the user and established a session key between the GW and SN. The authors claimed that the scheme was safe against insider attacks, impersonation attacks, and parallel session attacks. However, in [27] the authors provided a detailed analysis of the scheme of Yoo et al. and proved that the scheme is susceptible to various attacks, including insider attacks, impersonation attacks, parallel session attacks, password guessing attacks, fake registration attacks, and DOS attacks. Kumar et al. [36] proposed an authentication protocol for WSNs and claimed it could satisfy all the security requirements of the WSN; however, He et al. [37] proved that the scheme was weak against insider attacks and offline password guessing attacks and could not provide user anonymity. Kumar proposed another enhanced scheme in [13] and once again claimed that the scheme could withstand most of the known attacks and provide user privacy. However, with the Scyther implementation, given in Section 5.3, we found that the improved scheme presented in [13] was still vulnerable to insider attacks, parallel session attacks, and impersonation attacks. Farash et al. [15] proposed a key agreement and authentication protocol for WSNs in the Internet of Things (IoT) environment. The scheme was well designed and provided security against several well-known attacks. The author proved the strength of the protocol with BAN logic and further confirmed the theoretical analysis results by implementing the protocol in the AVISPA [38] tool. However, similar to other schemes discussed above, Farash et al. [15] did not consider the requirement of concurrent sessions, which are more likely to occur in an IoT environment. Moreover, our implementation of the scheme of Farash et al. [15] in Scyther revealed that the GW was vulnerable against insider attacks and impersonation attacks. The scheme was insecure in the presence of an intruder as discussed in Section 5.3, which assumed that the initial knowledge set of intruders included the identities of all sensor and user nodes. With known identities, an intruder can impersonate the user node and deceive the GW to falsify the authentication properties. Similarly, the Scyther implementation of the recently published work of Y. Lu et al. [16] and Quan et al. [14] revealed that at least two protocol participants falsified the authentication properties. The majority of the schemes discussed above provided the GW and user authentication schemes; however, Farash et al. [15] and Y. Lu et al. [16] also considered the GW and sensor node authentication, while Farash et al. [15] also allowed the user to access the data from sensor nodes directly. Li et al. [39] proposed an authentication protocol for sensor and user nodes. However, the proposed scheme requires time synchronization among protocol participants, and also employs the asymmetric elliptic curve cryptography, which is not a good design choice for resource limited WSN applications. For instance, very efficient public key algorithms, such as ECC [1], need a fraction of a second to execute the encryption/decryption procedures, while a symmetric key algorithm , such as RC5 [2], needs only a fraction of a millisecond to perform encryption and decryption procedures [3,4,5]. Unlike all the schemes discussed above, our proposed scheme, the SMSN authentication protocol suite, allows the sensor and user nodes to establish multiple concurrent connections with different sensor and sink nodes, which makes our scheme suitable for deploying it in the future to support IoT and related emerging technologies. Moreover, the SMSN authentication protocol suite provides several kinds of mutual authentications; for instance, after the initial authentication, a sensor or the user node receives an authentication ticket issued by the base station. The ticket can be further used for sensor-sink, user-sink, and user-sensor mutual authentication. The SMSN protocol suite consists of six protocols: three protocols deal with mobile sensor node authentication with sink nodes, and the other three deal with user node activation and authentication with the base station, sink nodes, and sensor nodes.

## 3. System Overview and Problem Statement

A typical WSN consists of the base station (*B*S), sink node (*S*), sensor node (*N*) and user node ($U_{i}$). We assume that the BS knows the public keys of the sink (*S*), sensor (*N*) and user (U) nodes.

### 3.1. System Architecture

An IoT smart service provider deploys the WSN with various base stations connected to the internet through a service center (SC). In the WSN, each $BS_{j}$ forms a group $G_{j}$ consisting of neighbor base stations and associated sink nodes. All group members share a symmetric group key $K_{G}^{j}$, which is controlled by the group master $BS_{j}$ using a group key agreement protocol such as discussed in [40]. Furthermore, the base station can access and download the profile of the mobile Sensor (*N*) and User (U) nodes. Each profile has a unique secret number $n_{s}^{i}$ ; besides being know to the base station, this secret number is also known to the corresponding Sensor (*N*) and User (U) nodes. The profile and unique secret number $n_{s}^{i}$ of all legitimate users and sensor nodes are accessible to the base station through the service center.

An example of the overall system architecture is depicted in Figure 1a. $BS_{1}$ creates a group consisting of neighbor $BS_{0}$ and $BS_{2}$ and associated sinks $\left(S_{1},S_{3}\right)$. $BS_{1}$ generates a group key $K_{G}^{1}$ and shares it among all group members. The profiles and the associated unique secret number of $U_{1},N_{1},$ and $N_{2}$ are accessible to $BS_{0},BS_{1},$ and $BS_{2}$ via the service center. An application scenario is given in Figure 1b. In a smart hospital, the health status of a hospitalized patient is continuously monitored by the several sensors mounted over the patient’s body. For the secrecy of patients’ health data it is essential that only authorized users be able to access the data. When a patient takes a walk in the hospital, all the mounted sensors continuously transfer the different sets of data to the nearby sinks. With the SMSN, the sensors do not need to follow the full authentication procedure for each data session; instead, the session continues by sharing a simple re-authentication ticket that provides the sensor-sink mutual authentication. Similarly, when an authorized doctor or nursing staff visits patients, he or she can access the sensor data in real time via a user device by establishing multiple data sessions with various sinks and sensor nodes. While he moves from patient to patient, his device does not need to follow a complete authentication procedure for each data session; rather, a simple re-authentication ticket can be used for re-authentication. From outside of the hospital, an authorized person can log into the hospital system and access the necessary data from sink and base stations by using the same re-authentication ticket.

### 3.2. Problem Statement

As described earlier, in IoT and other related emerging technologies, a mobile sensor or user node may need to exchange data with multiple nodes and so will experience the authentication process multiple times. With multiple concurrent connections, the authentication process becomes even more expensive while a node moves across the network. As shown in Figure 1a, sensor node $N_{1}$ is communicating with sink nodes $S_{5}$ and $S_{6}$ simultaneously. Likewise, user node $U_{1}$ is communicating with sink node $S_{3}$ and sensor node $N_{2}$ simultaneously. In such an application scenario, when a sensor or the user node moves across the network the frequency of the re-authentication process increases in proportion to the number of concurrent connections. Moreover, to establish multiple data sessions, it is essential that a protocol participant run multiple instances of the protocol run, which makes the security issue even more challenging. To perform multiple parallel re-authentications, it is evident that the protocol participants run multiple instances of the protocol. With the multiple protocol runs, the assurance of security of the protocol becomes more challenging. Therefore, for seamless services, lightweight yet secure re-authentication is vital.

According to our knowledge, we are the first to propose an authentication protocol for concurrent secure connections. To perform multiple parallel re-authentications, the protocol participants must run multiple instances of the protocol. With multiple protocol runs, the assurance of security of the protocol becomes more challenging. We developed our scheme, the SMSN, considering the following constraints: (1) the communication channels are insecure; (2) an intruder with the capabilities as described in Section-5-C is present in the network to launch various attacks; (3) due to the requirements for a WSN deployed in an IoT environment, the protocol participants are allowed to run multiple instances of the protocol; and (4) user and sensor nodes can dynamiclly leave and join the network and can move across the network.

### 3.3. Notations

- $BS_{j}$ = The *j*th base station
- $S_{j}$ = The *j*th sink
- $N_{i}$ = The *i*th sensor node
- $U_{i}$ = The *i*th user node
- A∼B = A is associated with B such that B is controlling authority
- $G_{j}$ = Group of all associated entities of *j*th BS
- $G_{j}^{o}$ = Group of all non-associated entities of *j*th BS who knows the $K_{G}^{j}$
- $K_{i}^{j}$ = The time-based key generated at *i*th interval by $BS_{j}$
- $K_{G}^{j}$ = A group key generated by $BS_{j}$
- $K_{S}^{i}$ = N→S session key generated at *i*th interval
- $K_{PS}^{i}$ = N→S private session key
- $K_{TS}^{i}$ = Temporary session key
- $E_{B}^{i}\left(m\right)$ = Encryption of ’m’, using key $K_{B}^{i}$
- $V_{i}$ = The index value for interval i
- $T_{k}$ = The *k*th Ticket
- Z(A) = An intruder *Z* mimicking the entity *A*
- $n_{i}$ = *i*th nonce in a message exchange

## 4. Proposed Scheme

The SMSN protocol suite consists of two protocol suites, the Keying Protocol suite and an Authentication Protocol suite. The Keying Protocol suit further comprises a key agreement protocol, a key retrieval protocol (which is the same as the one employed in [10]), and a key management protocol; likewise, the authentication protocol further comprises six protocols, three dealing with mobile sensor node authentication with sink nodes and the other three dealing with user node activation and authentication with a base station, sink nodes, and sensor nodes in different scenarios. In subsequent sections, the SMSN protocol suite is described in detail.

### 4.1. Keying Protocol Suite

In key generation protocol a ’commitment key’ is generated by group participants (the base station and sink nodes) using an irreversible function similar to that as used in [10,41]. The ’commitment key’ is further used to drive multiple time-based keys; for instance, the ticket encryption key and session key between the sink and user/sensor are derived from the ’commitment key’.

#### 4.1.1. Key Agreement Protocol

**Figure 2:**
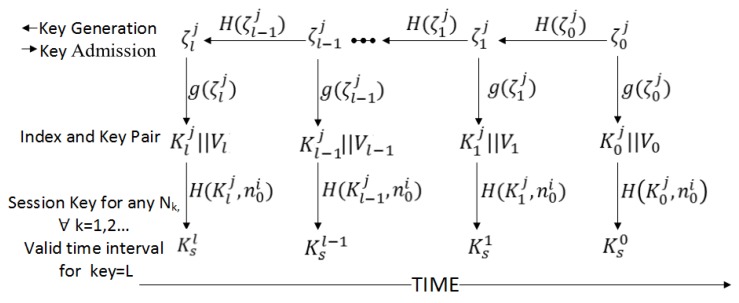
Time-based keys generation and admission with reference to time passage.

The key generation and distribution mechanism is shown in Figure 2 and consists of the following steps: (1) After every time interval $T_{d}$, $BS_{j}$ broadcasts the key generation information ($Key_{MSG}$) to all members of $G_{j}.$ (2) Using the key generation information ($KEY_{MSG})$, all members generate a Commitment Key Generator $\zeta_{0}^{j}=H\left(T_{d},N_{0}^{j}\right)$. (3) All members of $G_{j}$ can now generate a “Chain of Key Generators” of length *L* by using an irreversible one-way function: $H\left(\zeta_{0}^{j}\right)=\zeta_{1}^{j},H\left(\zeta_{1}^{j}\right)=\zeta_{2}^{j}\ldots H\left(\zeta_{l-1}^{j}\right)=\zeta_{l}^{j}$; i.e., $H{\left(\zeta_{k}^{j}\right)}^{i}=\zeta_{k+i}^{j}.$ (4) Each generator (ζ) in the chain is used by function *g* at specific intervals to derive indexes and a ticket encryption key pair. For instance, at interval *k* for any sensor or user node i, which is requesting the $BS_{j}$ to join the network, the function $g\left(\zeta_{k}^{j}\right)=H\left(\zeta_{k}^{j},H\left(n_{0}^{i}\right)\right)\left|\right|H\left(k\right)$ generates ticket encryption key $K_{k}^{j}$ and index $V_{k}$ value. In function *g* the value $n_{0}^{i}$ is a secret nonce sent by node *i* in a network join message. (5) Furthermore, $BS_{j}$ issues a session key based upon the ticket encryption key $K_{k}^{j}$ and secret nonce $n_{0}^{i}$, i.e., $K_{S}^{i}=H\left(K_{k}^{j},n_{S}^{i}\right)$ where $n_{S}^{i}$ is the secret random number assigned to legitimate sensor and user node.

All the symmetric keys generated in the above discussion have a size of 256 bits (32 bytes); hence, in the subsequent section of authentication protocols any symmetric encryption supporting the 256-bit key can be used, e.g., RC5/6 [2]; Rijndael [42], Twofish [43], MARS [44], and Blowfish [45].

#### 4.1.2. The Key Retrieval Protocol

After initial authentication, $BS_{j}$ issues a ticket to the requesting node. The ticket consists of two parts: (1) The first half consists of the sensor node identity (id) $N_{i}$, the session key $K_{s}^{i}$ , secret nonce $n_{0}^{i}$, and the profile. This part of the ticket is encrypted with time-based key $K_{l}^{i}$. (2) The second half consists of sensor node id $N_{i}$, the hash of group id $H(G^{j})$ , and the required information to retrieve the time-based key $K_{l}^{i}$. The hash of group id $H(G^{j})$ is an optional field used only if sink nodes can join multiple base stations; in that case, it is used to identify the group and to select the correct keychain. In the key retrieval, information depends on the selected mode and can be the scrambled index value *V*, index vector $V_{Hash}$, or index value *i*; the modes of a ticket are explained below.

**Mode-01:**

In mode-01, the ticket retrieval information comprises the index value $\left(V_{i}\right)$, requesting node id $N_{k}$, and the hash of the user’s private key. The ticket verifier searches the appended index value $\left(V_{i}\right)$ within its generated vector (V). A search hit at the ith place means that key $K_{i}^{j}$ can decrypt the ticket.

$$\begin{array}{cc} T_{k}=E_{i}^{j}(N_{i}\left|\right|K_{s}^{i}\left|\right|n_{0}^{i}\left||Prof|\right|E_{G}^{j}(N_{i}\left|\right|V_{i}||H\left(G^{j}\right)) & \left(\text{Ticket formate for mode-01}\right) \end{array}$$

**Mode-02:**

If the group members are in a fully secure environment, the SMSN employs a simple ticket retrieval strategy. Instead of using a scrambled index value *V* in mode-02 in the ticket retrieval, the information contains the interval value. In this mode, the ticket verifier does not need to run a search algorithm.

$$\begin{array}{cc} T_{k}=E_{i}^{j}(N_{i}\left|\right|K_{s}^{i}\left|\right|n_{0}^{i}\left||Prof|\right|E_{G}^{j}(N_{i}||i||H\left(G^{j}\right)) & \left(\text{Ticket formate for mode-02}\right) \end{array}$$

**Mode-03:**

**Figure 3:**
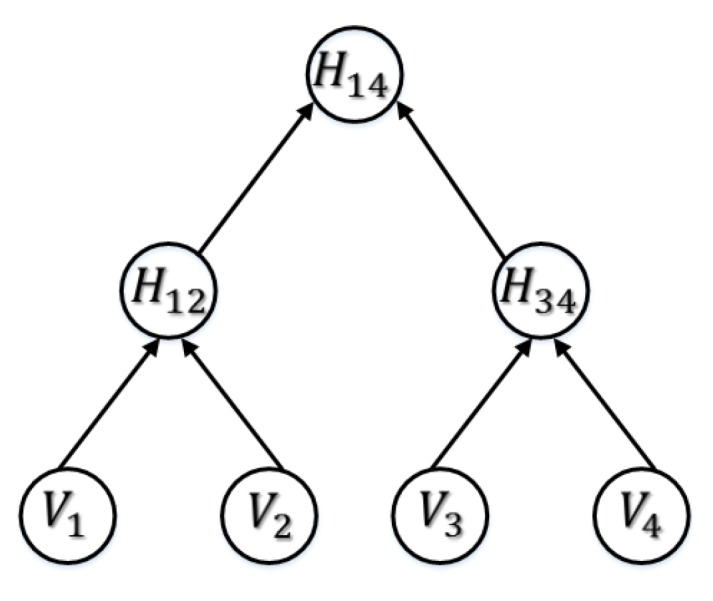
Example of Binary Hash Tree generated with index vector $V=v_{1},v_{2},v_{3},v_{4}$.

In mode-03, the ticket retrieval information is the same as in mode-01 except it includes a hash vector of size $\log_{2}\left|V\right|-2$. These hash values are carefully chosen nodes of the binary hash tree, which is generated such that the leaf nodes are indexed vector (V) values as shown in Figure 3.

$$\begin{array}{cc} T_{k}=E_{i}^{j}(N_{i}\left|\right|K_{s}^{i}\left|\right|n_{0}^{i}\left||Prof|\right|E_{G}^{j}(N_{i}\left|\right|V_{Hash}||H\left(G^{j}\right)) & \left(\text{Ticket formate for mode-03}\right) \end{array}$$

*Search Algorithm:*

- Start from the root and move down
- Ignore appended values and follow the path of the reconstructed node
- Continue until level $\log_{2}\left|V\right|-1$ is reached
- At level $\log_{2}\left|V\right|$, select the appended value that is the index value.

Mode-03 is suitable if the Chain of the Key Generator is very long. The tree “root node” is included in $T_{k}$ (optionally), which ensures that a trusted group member generated $T_{k}$.

#### 4.1.3. Key Management Protocol for the Sink Node

**Figure 4:**
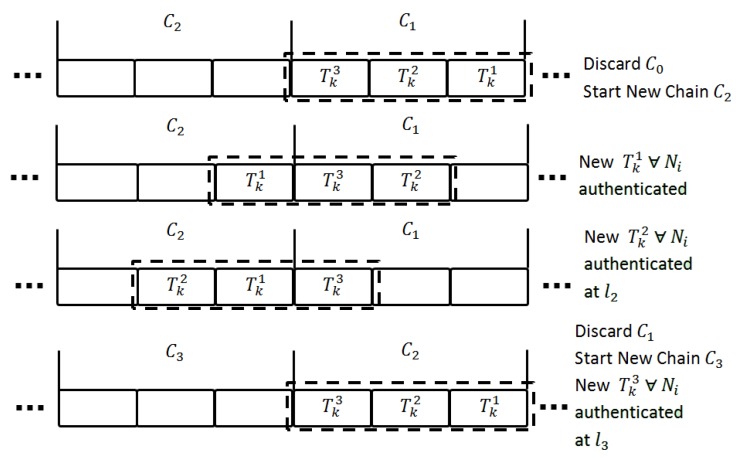
Managing the chain of the key generator in the sink.

At the start of the Chain of the Key Generator, the sink node reissues the ticket and session keys to associated nodes. To spread out the workload in the time dimension the sink node keeps the history of the previous key chain and issues a ticket/session key based on the moving window algorithm. Let us consider a chain with a length of 3; Figure 4 shows how the sink node spread the workload throughout the chain by adopting the moving window approach.

Step 1: The sink node discards the previous chain $C_{0}$ and generates the commitment key generator $G_{0}^{j}$ for the next chain $C_{2}$. Step 2: The sink node reissues the ticket and session key to all sensor nodes authenticated at interval 1. Step 3: The sink node reissues the ticket and session key to all sensor nodes authenticated at interval 2. Step 4: The sink node reissues the ticket and session key to all sensor nodes authenticated at interval 3 and discards the previous chain and generates the commitment key generator $G_{0}^{j}$ for the next chain.

### 4.2. Authentication Protocol Suite

When a sensor node joins the system, it goes through the Sensor Activation and Authentication Protocol (SAAP). After SAAP, $N_{i}$ can establish multiple concurrent secure connections with sink nodes using the authentication ticket ($T_{k}$); similarly, $N_{i}$ uses the $T_{k}$ for re-authentication while moving across the network. Likewise, when a user node $U_{i}$ joins the system, it goes through User Activation and Authentication Protocol (UAAP); subsequently, $U_{i}$ can use the authentication ticket ($T_{k}$) for authorization to collect data from multiple sink and sensor nodes. In a concurrent run of multiple instances of the protocol, the message authentication plays a critical part in preventing the replay attack and to achieve the objectives of the authentication protocol as defined in [19,20]. In an SMSN message, authentication is accomplished by a secure exchange of a randomly generated nonce challenge. Moreover, in all the protocols discussed below, if the protocol initiator (user or sensor node) does not hear the response to an authentication/switch request, the protocol initiator resends the authentication/switch request including a new nonce and ’resend’ flag. This step helps detect the impersonation, replay, and parallel session attack.

#### 4.2.1. Sensor Activation and Authentication Protocol (SAAP)

**Figure 5:**
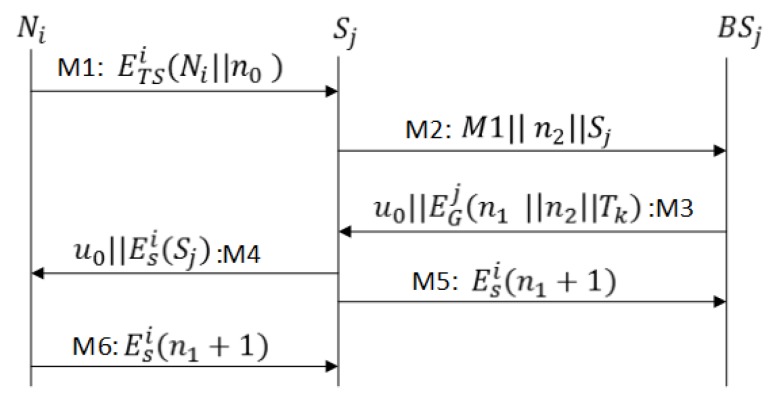
Message exchange for Sensor Activation and Authentication Protocol (SAAP) .

Sink node $S_{j}$ periodically broadcasts a Hello message ($BS_{j}\left|\right|S_{j})$. If a node $N_{i}$ wants to join the WSN, upon hearing the $Hellomessage,\;N_{i}$ generates an encryption key $K_{TS}^{i}=H\left(BS_{j}⊕n_{S}^{i}\right)$, encrypts the joining message with the generated key, and continues as follows:

M1 As shown in Figure 5, $N_{i}$ sends a JOIN message to sink $S_{j}$ enclosing $n_{0}$ and encrypted with the generated encryption key $K_{TS}^{i}$.
M2 Upon receiving the request from $N_{i}$, the $S_{j}$ forwards the request in conjunction with its identity and challenge $n_{2}$ to base station $BS_{j}$.
M3 $BS_{j}$ retrieves the profile from the database, and if $N_{i}$ isa legitimate sensor node, $BS_{j}$ generates the key $K_{TS}^{i}=H\left(BS_{j}⊕n_{S}^{i}\right)$, and sends $u_{0}=E_{TS}^{i}$ $\left(n_{0}+1||n_{1}||T_{k}||T_{R}||K_{s}^{i}\right)$ to $S_{j}$ in M3. M3 also includes ticket $T_{k}$, $n_{1}$ (a challenge for $N_{i}$), and $n_{2}$ (challenge response for the sink node), all encrypted with $K_{G}^{j}$. The sink node $S_{j}$ verifies the challenge $n_{2}$, stores $n_{1}$ and retrieves the profile and $K_{s}^{i}$ from the ticket.
M4 $S_{j}$ forwards the $u_{0}$ to $N_{i}.$ After a challenge ($n_{0}+1)$ verification, $N_{i}$ accepts $T_{k}$ and may start sending data to $S_{j}$.
M5 $S_{j}$ sends the challenge response $\left(n_{1}+1\right)$ to $BS_{j}$ for the confirmation of a successful protocol run.
M6 After challenge ($n_{1}+1)$ confirmation, $S_{j}$ starts accepting sensor data; otherwise, it marks $T_{k}$ as an invalid ticket.

In the SAAP, a secure exchange of $n_{0}$ ensures the message authentication between the sensor node and the base station, $n_{2}$ between the sink node and base station, and $n_{1}$ between the base station and the sink node, while message authentication between the sensor and sink nodes is established by session key encryption and $n_{1}$. If $N_{i}$ is already registered with $BS_{j}$, in M1 $n_{0}$ can be replaced with the hash of the password value.

#### 4.2.2. Sensor Re-Authentication Protocol -1 (SRP1)

**Figure 6:**
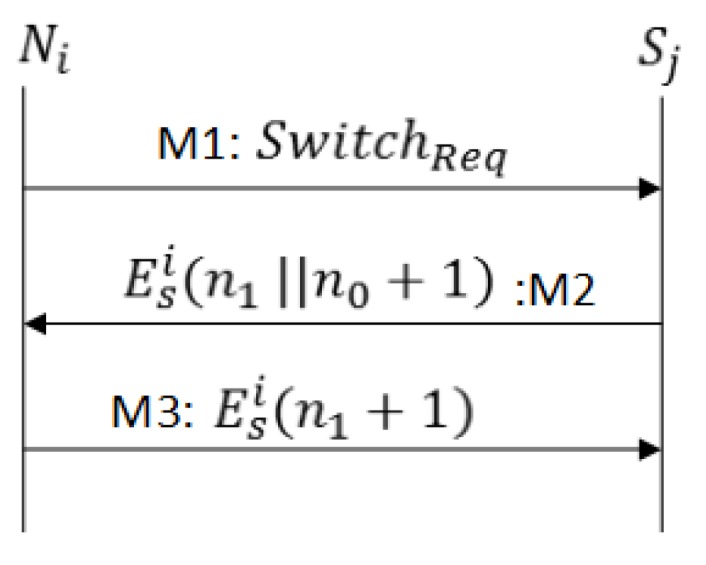
Message exchange for Sensor Re-Authentication Protocol-1 (SRP1).

If sensor node $N_{i}$ wants to establish multiple secure connections with sinks $S_{j}\in G_{i}$ or when a $N_{i}$ moves from $S_{k}\to S_{j}$ such that $\left\{S_{k},S_{j}\right\}\in G_{i}$, the Re-Authentication Protocol -1 continues as follows:

M1 As shown in Figure 6, $N_{i}$ sends $Switch_{Req}=E_{S}^{i}(N_{i}||H\left(n_{0}^{i}\right))||T_{k}$ to $S_{j}$. The sink $S_{j}$ decrypts the ticket, retrieves $K_{s}^{i},$ calculates the hash of $n_{0}^{i}$, and makes a comparison with $H(n_{0}^{i})$ received in the switch message. If the value $H(n_{0}^{i})$ does not match, the $S_{j}$ will ignore the request and otherwise proceed as follows.
M2 $S_{j}$ sends a challenge response along with the new challenge encrypted with session key $K_{s}^{i}$.
M3 $N_{i}$ sends the challenge response $n_{1}$. After challenge confirmation, $S_{j}$ starts accepting data; otherwise, it marks $T_{k}$ as an invalid ticket.

In the above procedure, secure exchange of $n_{0}^{i}$ ensures the message authentication between the sensor node and the sink node. With this feature, a sensor node can establish multiple secure sessions with various sink nodes as shown in Figure 1.

#### 4.2.3. Sensor Re-Authentication Protocol -2 (SRP2)

**Figure 7:**
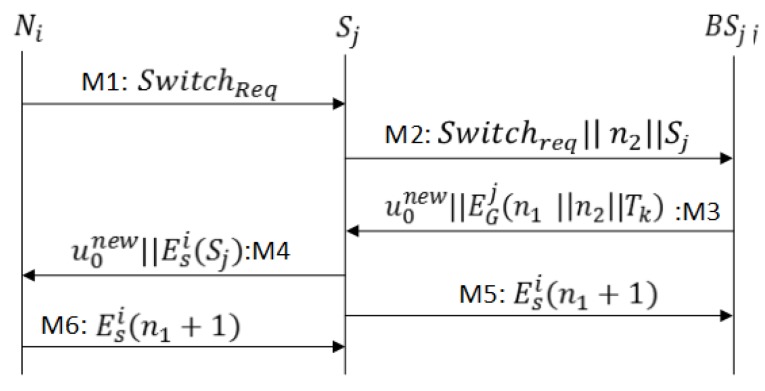
Message exchange for Sensor Re-Authentication Protocol-2 (SRP2).

If sensor node $N_{i}$ wants to establish another secure connection with sinks $S_{j}\in G_{k}^{o}$ or when a $N_{i}$ moves from $S_{k}\to S_{j}$ such that $S_{j}\in G_{k}^{o}$, the re-authentication procedure proceeds as follows:

M1 As shown in Figure 7, $N_{i}$ sends $Switch_{Req}=E_{S}^{i}(N_{i}||H\left(n_{0}^{i}\right))||T_{k}$ to $S_{j}$. The sink $S_{j}$ decrypts the second half of $T_{k}$ and verifies the identities of $N_{i}$ and ticket granting base station.
M2 After identities verification the sink $S_{j}$ forwards the request in conjunction with a challenge $n_{2}$ to base station $BS_{j}$.
M3 $BS_{j}$ retrieves the profile from $T_{k}$, and if $N_{i}$ isa legitimate sensor node, the $BS_{j}$ generates the key $K_{TS}^{i}=H\left(BS_{j}⊕n_{S}^{i}\right)$, and sends $u_{0}^{new}=E_{TS}^{i}(H\left(n_{0}^{i}\right)\left|\right|n_{1}\left|\right|T_{k}\left|\right|T_{R}\left|\right|K_{s}^{i})$ to $S_{j}$ in M3. M3 also includes ticket $T_{k}$, $n_{1}$ (a challenge for $N_{i}$), and $n_{2}$ (challenge response for the sink node), all encrypted with $K_{G}^{j}$. The sink node $S_{j}$ verifies the challenge $n_{2}$, stores $n_{1}$ and retrieves the profile and $K_{s}^{i}$ from the ticket.
M4 $S_{j}$ forwards $u_{0}^{new}$ to $N_{i}.$ After challenge ($n_{0}+1)$ verification $N_{i}$ accepts $T_{k}$ and start sending data to $S_{j}$.
M5 $S_{j}$ sends the challenge response $\left(n_{1}+1\right)$ to $BS_{j}$; it confirms a successful protocol run.
M6 After challenge ($n_{1}+1)$ confirmation $S_{j}$ start accepting data; otherwise, it marks $T_{k}$ as an invalid ticket.

In the SRP2, a secure exchange of $n_{0}$ ensures the message authentication between the sensor node and the base station, $n_{2}$ between the sink node and base station, and $n_{1}$ between the base station and the sink node, while message authentication between the sensor and sink nodes is established by session key encryption and $n_{1}$. If $N_{i}$ wants to share data in a secret mode, both $N_{j}$ and $S_{j}$ generate a private session key $K_{sp}^{j}=H\left(K_{s}^{i},n_{0}^{i},n_{1}\right)$. With this feature, a sensor node can establish multiple secure sessions with various sink nodes as shown in Figure 1.

#### 4.2.4. User Activation and Authentication Protocol (UAAP)

**Figure 8:**
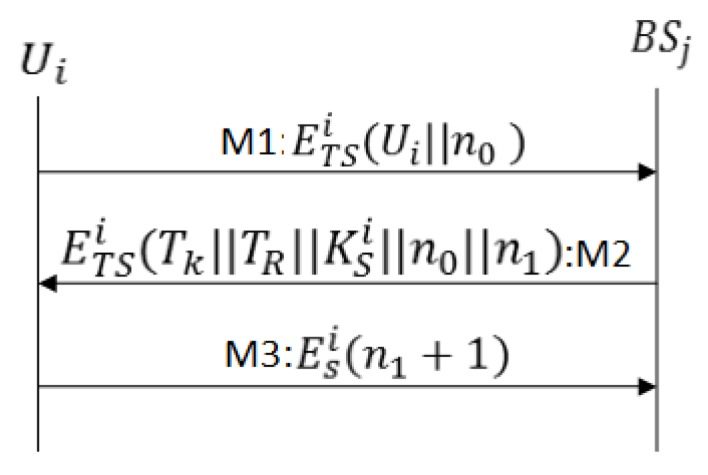
Message exchange for User Activation and Authentication Protocol (UAAP).

In some scenarios a user may desire to access data from the sensor network, for example, in IoT applications such as smart homes and smart buildings, a smartphone user may want to get sensor node data. In SMSN authentication protocol suites, an authenticated user (holding a valid ticket) can access data directly from sensor nodes and/or can collect from the sink node. The ticket structure for $U_{i}$ is the same as the ticket structure discussed above for $N_{i}$ where the sensor node identity and profile are replaced with the user node identity and profile. The user profile information includes the permissible accessibility information. In the User Activation and Authentication Protocol (UAAP) a user can acquire a ticket from $BS_{j}$ in two different ways. If the user is not in the communication range of $BS_{j}$ it routes the joining message via the nearest sink node $S_{j}$; the protocol proceeds exactly as in the SAAP. However, if the user is in the communication range of $BS_{j}$, the user $U_{i}$ generates an encryption key $K_{TS}^{i}=H\left(BS_{j}⊕n_{S}^{i}\right)$, encrypts the joining message with the generated key, and proceeds as follows:

M1 As shown in Figure 8, $U_{i}$ sends a JOIN message enclosing $n_{0}$ and the user identity to base station $BS_{j}$.
M2 $BS_{j}$ retrieves profile from the database, and if $U_{i}$ is a legitimate user, the $BS_{j}$ generates authentication ticket, and send along with, $n_{1}$ ( a challenge for $U_{i}$), and $n_{0}$ (challenge response), all encrypted with $K_{TS}^{i}=H\left(BS_{j}⊕n_{S}^{i}\right)$. The ticket structure is same except the secret nonce $n_{1}$ enclosed inside ticket is generated by $BS_{j}.$
M3 The user node $U_{i}$ verifies the challenge $n_{0}$, stores $T_{k}$ and $n_{1}$. $U_{i}$ sends the challenge response $\left(n_{1}+\;1\right)$ to $BS_{j}$; it confirms a successful protocol run . After receiving a challenge response $\left(n_{1}+1\right)$ the $BS_{j}$ updates the status of $U_{i}$ from idle to active user.

In User Activation and Authentication Protocol, secure exchange of $n_{0}$ ensures the message authentication between user node and base station and $n_{1}$ between the base station and user node.

#### 4.2.5. User-Sink Authentication Protocol (USiAP)

After acquiring the authentication ticket, if user $U_{k}$ wants to retrieve data from sink nodes $S_{j}$, it sends a JOIN request in conjunction with a ticket to $S_{j}$ and then follows the same procedure as discussed in Sensor Re-Authentication Protocols 1 and 2; except after ticket verification, $S_{j}$ can piggyback data with the rest of the messages. Using the authentication ticket, user $U_{k}$ can also establish multiple concurrent connections with various sink nodes.

#### 4.2.6. User- Sensor Authentication Protocol (USeAP)

**Figure 9:**
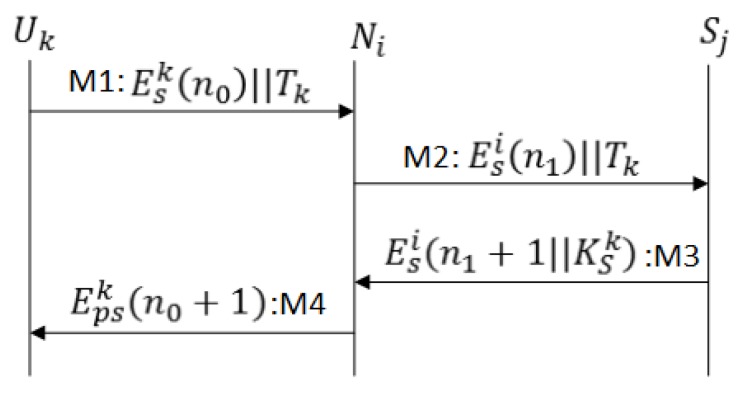
Message exchange for User-Sensor Authentication Protocol (USeAP).

After acquiring the authentication ticket, if user $U_{k}$ wants to access data directly from sensor nodes $N_{i}$, it sends a JOIN request in conjunction with a ticket to $N_{i}$ and the procedure proceeds as follows:

M1 As shown in Figure 9, $U_{k}$ sends a JOIN message in conjunction with ticket and challenge $n_{0}$ encrypted with its ticket centered session key.
M2 Upon receiving the request from $U_{k}$ the $N_{i}$ forward the ticket and encrypted challenge $n_{1}$ to sink node $s_{j}.$
M3 Sink decrypts the ticket and retrieves $U_{k}^{\prime }$s profile and session key $K_{S}^{k}$, and if $U_{i}$ is a legitimate user node, $S_{j}$ sends $K_{S}^{k}$ and challenge response $\left(n_{1}+1\right)$ to $N_{i}$.
M4 After challenge verification, $N_{i}$ generates a private session key $K_{ps}^{k}=H\left(K_{s}^{k},n_{0}\right)$ and sends a challenge response encrypted with the private session key. $U_{k}$ also generates the private session key and verifies the challenge response.

In User-Sensor Authentication Protocol suite, secure exchange of $n_{0}$ ensures the message authentication between users and sensor node, while the secure exchange of $n_{1}$ ensures the message authentication between sensor node and sink node.

## 5. Security Analysis

This section presents the comprehensive security analysis of the SMSN protocol, including an informal security analysis and discussion of a formal security analysis using BAN logic [32], and finally presents the Scyther [17,18] implementation result of the SMSN and previously proposed schemes [11,12,13,14,15,16].

### 5.1. Informal Analysis and Discussion

To verify the strength of the SMSN protocol against known attacks we introduce an intruder in the network with capabilities as follows: It has an initial information set that contains the IDs of all users, sensor nodes, sink nodes and base stations. It can intercept and record message exchanges between participating entities. It can redirect, spoof, and replay the messages. The subsequent sections show that the intruders, with all the above-mentioned capabilities, fail to launch a successful replay, parallel session, man-in-middle , impersonation, and several other attacks against the SMSN protocol suite.

#### 5.1.1. Replay, Multiplicity, Parallel and Man in Middle Attacks Against the SMSN

**Figure 10:**
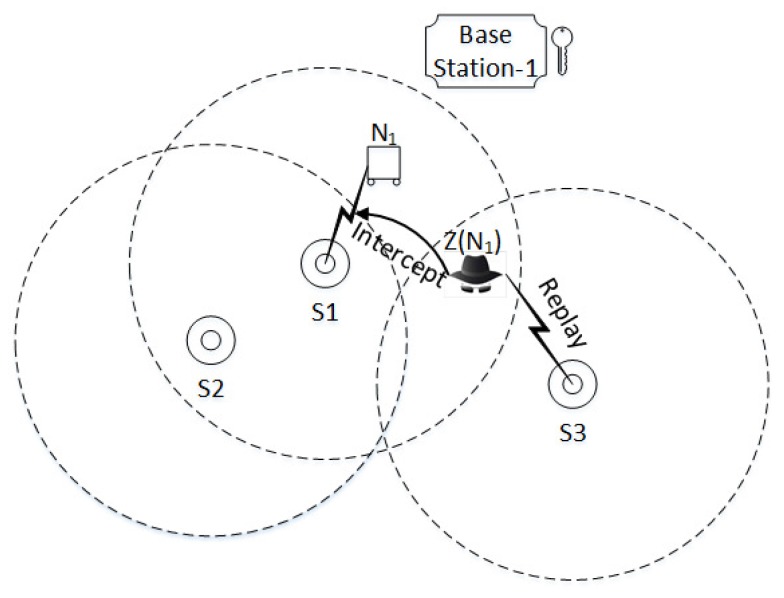
The intruder *Z* impersonates the protocol initiator $N_{1}$ (can be a sensor or user node). Intruder $Z(N_{1})$ intercepts the messages between $N_{1}$ and sink S1 and replays them to sink S2. Both sinks are associated with base station 1 and share the same keychain.

**Figure 11:**
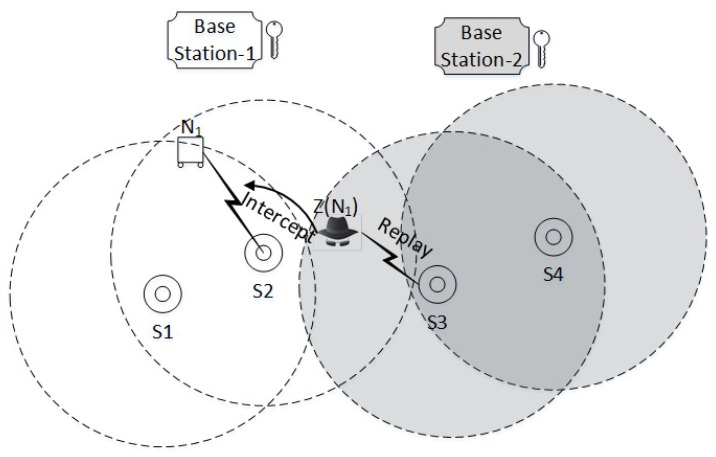
The intruder *Z* impersonates the protocol initiator $N_{1}$ (can be a sensor or user node). Intruder $Z(N_{1})$ intercepts the messages between $N_{1}$ and sink S2 and replays them to sink S3. Sink S2 is associated with base station 1 , and Sink S3 is associated with base station 2.

**Figure 12:**
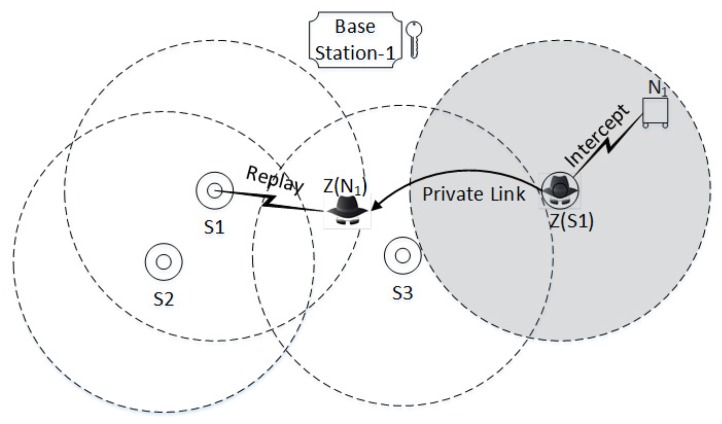
Two intruders impersonate the sink node $S_{1}\;BS_{1}$ and sensor node $N_{1}$; the intruder $Z(N_{1})$ is within the region of $S_{1}\;BS_{1}$, and intruder $Z(S_{1})$ is outside somewhere close to node $N_{1}$. Both intruders can communicate with zero communication delay via a private link.

We introduce an intruder, as discussed above, in the network and launch replay, multiplicity, parallel session, and man-in-middle attacks against the SMSN for three different scenarios, as shown in Figure 10, Figure 11 and Figure 12. The intruder *Z*impersonates a protocol participant, intercepts the messages, and replays them to deceive other protocol participants. Replay, multiplicity, parallel, and man-in-middle attacks against the SAAP for three different scenarios are given below.

**Scenario 1:**

For the given scenario in Figure 10, let us suppose a sensor node $N_{i}$ sends a request for authentication to sink $S_{j}∼BS_{j}$ . During the protocol run an intruder $Z(N_{i})$ intercepts the messages and replays them to another sink $S_{i}∼BS_{j}$; the attack proceeds as follows: The intruder $Z(N_{i})$ intercepts M1 and replays it to $S_{i}$. The attack is detected immediately when $BS_{j}$ receives two M2 messages enclosing the same M1. $BS_{j}$ sends M3 to both sinks comprising the ’Alert’ flag and a different $n_{1}$ nonce challenge. $Z(N_{i})$ intercepts M6 and replays it to $S_{i}$; note that the intercepted message M6 comprises a different $n_{1}$ which is the only valid response for a sink $S_{j}$. Upon receiving the wrong challenge response, the sink $S_{i}$ identifies the intruder node. The SAAP not only detects the replay attack but also identifies the intruder.

**Scenario 2:**

For the given scenario in Figure 11, let us suppose a sensor node $N_{i}$ sends a request for authentication to sink $S_{j}∼BS_{j}$ . During the protocol run an intruder $Z(N_{i})$ intercepts the messages and replays them to another sink $S_{i}∼BS_{i}$; the attack proceeds as follows: The intruder $Z(N_{i})$ intercepts M1 and replays it to $S_{i}$. Unlike in scenario 1, neither base station $BS_{j}$ and $BS_{i}$ can detect the attack at this stage and replies with a normal M3 to associated sink nodes $S_{i}$ and $S_{j},$ respectively. However, both M3 messages comprise a different $n_{1}$ nonce challenge. $Z(N_{i})$ intercepts the M6 and replays it to $S_{i}$; note that the intercepted message M6 contains a different $n_{1}$ which is the only valid response for the sink $S_{j}$. Upon receiving the wrong challenge response, the sink $S_{i}$ identifies the intruder.

**Scenario 3:**

For the given scenario in Figure 12, let us suppose two intruders impersonate the sink $S_{j}∼BS_{j}$ and sensor node $N_{i}$; the intruder $Z(N_{i})$ is within the region of $S_{i}∼BS_{i}$ , and intruder $Z(S_{j})$ is outside somewhere close to node $N_{i}.$ Furthermore, both intruders can communicate through a private link with zero delay. During the protocol run intruder $Z(S_{j})$ intercepts the messages sent by sensor node $N_{i}$ and replays it to $BS_{j}$; also $Z(S_{j})$ shares all the intercepted messages with fellow intruder $Z(N_{i})$ via a private secure channel; the attack proceeds as follows: The intruder $Z(S_{j})$ intercepts M1 sent by $N_{i}$ and shares the intercepted message with $Z(N_{i})$. The intruder $Z(N_{i})$ sends the intercepted message to $S_{i}∼BS_{i}$; the protocol proceeds normally and upon receiving M4 the intruder $Z(N_{i})$ sends the message M4 to $Z(S_{j})$. The intruder $Z(S_{j})$ sends M4 to sensor node $N_{i}$; upon receiving M6 the intruder $Z(S_{j})$ shares the message M6 with $Z(N_{i})$. The intruder $Z(N_{i})$ sends M6 to $S_{i}∼BS_{i}$ and the attack is completed. The attack is only successful if the replay of M6 is delivered to $S_{j}$ within a time interval of Td/L where *L* is the length of the keychain. Moreover, in practice when the private link between fellow intruders adds a communication delay, $N_{i}$ can detect the attack by comparing the TR (registration time sent in $u_{0})$ with the local time.

As the outcome of the above attack, $N_{i}$ considers that the authentication process was completed successfully. However, the intruders cannot get any useful information during a protocol run: $Z(S_{j})$ does not know the session key delivered to $N_{i}$, so $Z(S_{j})$ cannot further communicate with $N_{i}$. Due to the unavailability of a data link, $N_{i}$ uses the ticket to run SRP1/2. However, a problem exists on the other side: sink $S_{i}$ and base station $BS_{i}$ consider that they authenticated a legitimate sensor $N_{i}$ successfully; from their point of view, the sensor $N_{i}$ is within the region of $BS_{i}$ but in reality, $N_{i}$ is in the region of $BS_{j}$. Similarly, in the case of SRP1, SRP2, UAAP, USiAP, and USeAP the intruder *Z* fails to launch successful attacks for scenarios 1 and 2; however, the intruder *Z*completes the attack in scenario 3.

In a nutshell, replay, multiplicity, parallel and man-in-middle attacks against the SMSN protocol are not successful. Even though in scenario 3 the intruders completed the attack, they could not cause a serious security issue because after the completion of the protocol run, the intruder could not get useful information such as the session key, ticket, or partial session key. Furthermore, the attack can be avoided by introducing a timestamp in each message exchange. For illustration, the intruders need to wait for a significantly longer time to replay M4 and M6, and this significant delay can be detected with the time stamp, which reveals the existence of the intruder in the network.

#### 5.1.2. Black Hole Attack

In a black hole attack [46,47,48] the intruder impersonates a node and blocks or drops the messages upon receiving them. In the SMSN, the sensor and user nodes can connect to multiple sink nodes simultaneously; hence, failure of data exchange on one route does not block the data delivery towards the base station. Moreover, the black hole attack is detectable in our scheme because the SMSN ensures binding by employing an exchange of secret nonce between $N_{i}↔S_{j},\;N_{i}↔BS_{j}$, and $B_{j}↔B.$ Consecutive failures of exchange of challenge detects the black hole attack. Once the black hole is detected, the sensor node can send data via another sink node.

#### 5.1.3. Wormhole Attack

In a wormhole attack [49,50], the intruder captures the messages in one location and tunnel to another location to a fellow intruder who replays the tunneled messages in another location area. The attack discussed in scenario 3 can be regarded as a wormhole attack. From the point of view of replay, multiplicity, parallel and man-in-middle attacks, the attack in scenario 3 did not achieve its objectives, but from the point of view of the wormhole attack, the attack is successful. The solution for the problem is similar to the one we discussed earlier: the attack can be avoided by introducing a timestamp in each message exchange.

#### 5.1.4. Analytical Attacks

In an analytic attack [31,51], the intruder intercepts the messages and using cryptanalysis tries to recover a cryptographic key. With the inclusion of a time-based key, our scheme inherits the freshness property, which defies the capability of the intruder to launch analytical attacks, as it has a max time of $T_{d}$ to acquire the time-based key, which makes it difficult to launch analytical attacks.

#### 5.1.5. Topological Centered Attacks

In [52,53] the authors presented an authentication protocol for a sensor network in which the sink issues a re-authentication ticket that includes a list of neighbor sink nodes. This information can lead to topological centered attacks [27,28] such as identity replication attacks. In our scheme, the topological information is entirely obscured from the sink and sensor nodes.

### 5.2. Formal Analysis Using BAN Logic

The BAN logic [32] is a widely used formal method for the formal analysis of security protocols. To prove the security of the SMSN protocol suite it is sufficient to demonstrate the security of the SAAP and UAAP protocols; the rest of the protocols are extensions of the SAAP and UAAP and use the ticket and session key established in the SAAP and UAAP protocols run. Hence, proof of SAAP and UAAP protocols concludes the security of the SMSN protocol suite.

The three basic objects of BAN logic are principals, formula/statements, and encryption keys. The principals, the protocol participants, are represented by symbols *P* and *Q*. The formula/statements are symbolized by *X* and *Y* and represents the content of the message exchanged. The encryption keys are symbolized by *K*. The logical notations of BAN-logic used for our analysis is given below:

- P⊧X:P believes *X*, or *P* would be enabled to believe *X*; in conclusion, *P* can take *X* as true.
- P≺X:P sees/receives *X*. *P* initially has or received a message *X* and *P* can see the contents of the message and is capable of repeating *X*.
- P|∼X:P once said X. *P* has sent a message including the statement *X*. However, the freshness of message is unknown.
- P⇒X:P controls *X* and should be trusted for formula/statement *X*.
- #(X):X is fresh; it says, *X* never sent by any principal before.
- $P\overset{K}{↔}Q:P$ and *Q* shares a key *K* to communicate in a secure way and *K* is only known to *P*, *Q* and a trusted principal.
- ${\left(X\right)}_{K}:$ The statement *X* is encrypted by key K.
- ${\left\{X\right\}}_{Y}:$ It stand for *X* combined with Y. *Y* is anticipated to be secret and its implicit or explicit presence proves the identity of a principal who completes the ${\left\{X\right\}}_{Y}$.

Some primary BAN-logic postulates used in the analysis of the SMSN are given below:

- Message meaning rules: $\frac{P⊧P\overset{K}{↔}Q,P≺{\left(X\right)}_{K}}{P⊧Q|∼X}$, $\frac{P⊧P\overset{Y}{↔}Q,P≺X_{Y}}{P⊧Q|∼X}$
- Nonce verification rule: $\frac{P⊧\#(X),P⊧Q|∼X}{P⊧Q⊧X}$
- Jurisdiction rule: $\frac{P⊧Q\Rightarrow X,P⊧Q⊧X}{P⊧X}$
- Freshness rule: $\frac{P⊧\#(X)}{P⊧(X,Y)}$
- Believe rule: $\frac{P⊧Q⊧(X,Y)}{P⊧X,P⊧Y}$
- Session key rule: $\frac{P⊧Q\#(X),P⊧Q⊧X}{P⊧P\overset{K}{↔}Q}$

#### 5.2.1. BAN Logic Analysis of SAAP

The SAAP protocol should achieve the following goals:

G1 $N_{i}⊧\left(N_{i}\overset{K_{S}^{i}}{↔}S_{j}\right)$
G2 $N_{i}⊧S_{j}⊧\left(N_{i}\overset{K_{S}^{i}}{↔}S_{j}\right)$
G3 $S_{j}⊧\left(N_{i}\overset{K_{S}^{i}}{↔}S_{j}\right)$
G4 $S_{j}⊧N_{i}⊧(N_{i}\left(N_{i}\overset{K_{S}^{i}}{↔}S_{j}\right)$

Protocol Idealization:

I1 $N_{i}\overset{ViaS_{j}}{↔}BS_{j}:{\left\{n_{0},\left(N_{i}\overset{n_{S}^{i}}{↔}BS_{j}\right)\right\}}_{H(X_{S})}$
I2 $S_{j}\to BS_{j}:\left\{n_{2},IDS_{j}\right\}$
I3 $BS_{j}\to S_{j}:\left\{{\left(n_{1},n_{2},{\left(N_{i}\overset{K_{S}^{i}}{↔}S_{j},\#\left(N_{i}\overset{K_{S}^{i}}{↔}S_{j}\right),n_{0}^{i}\right)}_{K_{l}^{i}=H\left(\zeta_{l}^{j},H\left(n_{0}^{i}\right)\right)},V_{i}\right)}_{K_{G}^{i}},{\left\{n_{0},n_{1},{\left(N_{i}\overset{K_{S}^{i}}{↔}S_{j},\#\left(N_{i}\overset{K_{S}^{i}}{↔}S_{j}\right),n_{0}^{i}\right)}_{K_{l}^{i}=H\left(\zeta_{l}^{j},H\left(n_{0}^{i}\right)\right)},T_{R},N_{i}\overset{K_{S}^{i}}{↔}S_{j},\#\left(N_{i}\overset{K_{S}^{i}}{↔}S_{j}\right),N_{i}\overset{n_{S}^{i}}{↔}BS_{j}\right\}}_{H(X_{S})}\right\}$
I4 $S_{j}\overset{ViaBS_{j}}{↔}N_{i}:{\left\{n_{0},n_{1},{\left(N_{i}\overset{K_{S}^{i}}{↔}S_{j},\#\left(N_{i}\overset{K_{S}^{i}}{↔}S_{j}\right),n_{0}^{i}\right)}_{K_{l}^{i}=H(\zeta_{l}^{j},H\left(n_{0}^{i})\right)},T_{R},N_{i}\overset{K_{S}^{i}}{↔}S_{j},\#\left(N_{i}\overset{K_{S}^{i}}{↔}S_{j}\right),N_{i}\overset{n_{S}^{i}}{↔}BS_{j}\right\}}_{H(X_{S})}$
I5 $S_{j}\to N_{i}:{\left(IDS_{j}\right)}_{K_{s}^{i}}$
I6 $N_{i}\to S_{j}:{\left(n_{1}\right)}_{K_{S}^{i}}$

Initial State Assumptions:

A1 $BS_{j}⊧\#\left(n_{0}\right)$
A2 $BS_{j}⊧\#\left(n_{2}\right)$
A3 $S_{j}⊧\#\left(n_{1}\right)$
A4 $N_{i}⊧\#\left(n_{1}\right)$
A5 $N_{i}⊧\left(N_{i}\overset{K_{TS}=H\left(X_{S}\right)}{↔}BS_{j}\right)$
A6 $BS_{j}⊧\left(N_{i}\overset{K_{TS}=H\left(X_{S}\right)}{↔}BS_{j}\right)$
A7 $S_{j}⊧\left(S_{j}\overset{K_{G}^{j}}{↔}BS_{j}\right)$
A8 $BS_{j}⊧\left(S_{j}\overset{K_{G}^{j}}{↔}BS_{j}\right)$
A9 $BS_{j}⊧N_{i}⊧\left(N_{i}\overset{K_{TS}=H\left(X_{S}\right)}{↔}BS_{j}\right)$
A10 $N_{i}⊧BS_{j}⊧\left(N_{i}\overset{K_{TS}=H\left(X_{S}\right)}{↔}BS_{j}\right)$
A11 $BS_{j}⊧S_{j}⊧\left(S_{j}\overset{K_{G}^{j}}{↔}BS_{j}\right)$
A12 $S_{j}⊧BS_{j}⊧\left(S_{j}\overset{K_{G}^{j}}{↔}BS_{j}\right)$

Let us analyze the protocol to show that $N_{i}$ and $S_{j}$ share a session key:

From I1, we have

(1) $$BS_{j}≺{\left\{n_{0},\left(N_{i}\overset{n_{S}^{i}}{↔}BS_{j}\right)\right\}}_{H(X_{S})}$$

The (1), A6 and message meaning rule infers that

(2) $$S_{j}⊧N_{i}|∼\left\{n_{0},\left(N_{i}\overset{n_{S}^{i}}{↔}BS_{j}\right)\right\}$$

The A1 and freshness conjuncatenation comprehends that

(3) $$BS_{j}⊧\#\left\{n_{0},\left(N_{i}\overset{n_{S}^{i}}{↔}BS_{j}\right)\right\}$$

The (2), (3) and nonce verification rule deduces that

(4) $$BS_{j}⊧\left\{N_{i}⊧n_{S}^{i},n_{0},\left(N_{i}\overset{n_{S}^{i}}{↔}BS_{j}\right)\right\}$$

The (4) and believe rule infers that

(5) $$BS_{j}⊧N_{i}⊧\left(N_{i}\overset{n_{S}^{i}}{↔}BS_{j}\right)$$

From A2, (5) and jurisdiction rule, it concludes

(6) $$BS_{j}⊧\left(N_{i}\overset{n_{S}^{i}}{↔}BS_{j}\right)$$

This belief confirms that $BS_{j}$ has received a message from a legitimate $N_{i}$.

From I2, we have

(7) $$BS_{j}≺n_{2}$$

The (7) and message meaning it infers that

(8) $$BS_{j}⊧S_{j}|∼n_{2}$$

The A2, A1, (3) and freshness conjuncatenation comprehends that

(9) $$BS_{j}⊧\#\left\{n_{0},n_{2},\left(N_{i}\overset{n_{S}^{i}}{↔}BS_{j}\right)\right\}$$

According to nonce freshness, this proves that $BS_{j}$ confirms that $N_{i}$ is recently alive and running the protocol with $BS_{j}$.

From I3, we have

(10) $$S_{j}≺{\left(n_{1},n_{2},{\left(N_{i}\overset{K_{S}^{i}}{↔}S_{j},\#\left(N_{i}\overset{K_{S}^{i}}{↔}S_{j}\right),n_{0}^{i}\right)}_{K_{l}^{i}=H\left(\zeta_{l}^{j},H\left(n_{0}^{i}\right)\right)},V_{i}\right)}_{K_{G}^{i}}$$

The A7 and (10) deduce that

(11) $$S_{j}⊧BS_{j}|∼\left\{n_{1},n_{0}^{i},N_{i}\overset{K_{S}^{i}}{↔}S_{j},\#\left(N_{i}\overset{K_{S}^{i}}{↔}S_{j}\right),V_{i}\right\}$$

The A3, (11) and freshness conjuncatenation comprehends that

(12) $$S_{j}⊧\#\left\{n_{1},n_{0}^{i},N_{i}\overset{K_{S}^{i}}{↔}S_{j},\#\left(N_{i}\overset{K_{S}^{i}}{↔}S_{j}\right),V_{i}\right\}$$

The (11), (12) and nonce verification rule infers that

(13) $$S_{j}⊧BS_{j}⊧\left\{n_{1},n_{0}^{i},N_{i}\overset{K_{S}^{i}}{↔}S_{j},\#\left(N_{i}\overset{K_{S}^{i}}{↔}S_{j}\right),V_{i}\right\}$$

The (13) and believe rule comprehends that

(14) $$S_{j}⊧BS_{j}⊧\left(N_{i}\overset{K_{S}^{i}}{↔}S_{j}\right)$$

The logic belief proves that $S_{j}$ is confident and believes that $K_{S}^{i}$ is issued by $BS_{j}$; moreover, the freshness of the key also suggests that $BS_{j}$ is alive and running the protocol with $S_{j}$ and $N_{i}$.

The (13), (14) and jurisdiction rule concludes that (15 Goal-3)

(15) $$S_{j}⊧\left(N_{i}\overset{K_{S}^{i}}{↔}S_{j}\right)$$

From I4, we have

(16) $$N_{i}≺{\left\{n_{1},,T_{R},N_{i}\overset{K_{S}^{i}}{↔}S_{j},\#\left(N_{i}\overset{K_{S}^{i}}{↔}S_{j}\right),N_{i}\overset{n_{S}^{i}}{↔}BS_{j}\right\}}_{H(X_{S})}$$

The (16), A5 and message meaning rule comprehends that

(17) $$N_{i}⊧BS_{j}|∼\left\{n_{1},,T_{R},N_{i}\overset{K_{S}^{i}}{↔}S_{j},\#\left(N_{i}\overset{K_{S}^{i}}{↔}S_{j}\right)\right\}$$

The (17), A4 and freshness conjuncatenation rule infers that

(18) $$N_{i}⊧\#\left\{n_{1},,T_{R},N_{i}\overset{K_{S}^{i}}{↔}S_{j},\#\left(N_{i}\overset{K_{S}^{i}}{↔}S_{j}\right)\right\}$$

The (17), (18) and nonce verification rule deduce that

(19) $$N_{i}⊧BS_{j}⊧\left\{n_{1},,T_{R},N_{i}\overset{K_{S}^{i}}{↔}S_{j},\#\left(N_{i}\overset{K_{S}^{i}}{↔}S_{j}\right)\right\}$$

The (19) and believe rule infers that

(20) $$N_{i}⊧BS_{j}⊧\left\{N_{i}\overset{K_{S}^{i}}{↔}S_{j},\right\}$$

The (19), (20) and jurisdiction rule concludes that (21 Goal-1)

(21) $$N_{i}⊧\left\{N_{i}\overset{K_{S}^{i}}{↔}S_{j},\right\}$$

From I5, we have

(22) $$N_{i}≺IDS_{j}$$

The (15), (21), (22) and meaning rule comprehends that (23 Goal-4)

(23) $$S_{j}⊧N_{i}⊧\left\{N_{i}\overset{K_{S}^{i}}{↔}S_{j},\right\}$$

From I6, we have

(24) $$S_{j}≺n_{1}$$

The (15), (21), (23) and nonce verification rule deduce that (25 Goal-2)

(25) $$N_{i}⊧S_{j}⊧\left\{N_{i}\overset{K_{S}^{i}}{↔}S_{j},\right\}$$

1. *BAN Logic Analysis of UAAPP:*

The UAAP protocol should achieve the following goals:

G1 $U_{i}⊧\left(U_{i}\overset{K_{S}^{i}}{↔}S_{j}\right)$
G2 $BS_{j}⊧U_{i}⊧\left(U_{i}\overset{K_{S}^{i}}{↔}S_{j}\right)$
G3 $U_{i}⊧BS_{j}⊧\left(U_{i}\overset{K_{S}^{i}}{↔}S_{j}\right)$

Idealization of UAAP:

I1 $U_{i}\to BS_{j}:{\left\{n_{0},\left(U_{i}\overset{n_{S}^{i}}{↔}BS_{j}\right)\right\}}_{H(X_{S})}$
I2 $BS_{j}\to U_{i}:{\left\{n_{0},n_{1},{\left(U_{i}\overset{K_{S}^{i}}{↔}S_{j},\#\left(U_{i}\overset{K_{S}^{i}}{↔}S_{j}\right),n_{0}^{i}\right)}_{K_{l}^{i}=H\left(\zeta_{l}^{j},H\left(n_{0}^{i}\right)\right)},{\left(V_{i}\right)}_{K_{G}^{i}},T_{R},N_{i}\overset{K_{S}^{i}}{↔}S_{j},\#\left(U_{i}\overset{K_{S}^{i}}{↔}S_{j}\right),U_{i}\overset{n_{S}^{i}}{↔}BS_{j}\right\}}_{H(X_{S})}$
I3 $U_{i}\to BS_{j}:{\left(n_{1}\right)}_{K_{S}^{i}}$

Initial State Assumptions of UAAP:

A1 $BS_{j}⊧\#\left(n_{0}\right)$
A2 $U_{i}⊧\#\left(n_{1}\right)$
A3 $N_{i}⊧\left(U_{i}\overset{K_{TS}=H\left(X_{S}\right)}{↔}BS_{j}\right)$
A4 $BS_{j}⊧\left(U_{i}\overset{K_{TS}=H\left(X_{S}\right)}{↔}BS_{j}\right)$
A5 $S_{j}⊧N_{i}⊧\left(N_{i}\overset{K_{TS}=H\left(X_{S}\right)}{↔}BS_{j}\right)$
A6 $N_{i}⊧BS_{j}⊧\left(N_{i}\overset{K_{TS}=H\left(X_{S}\right)}{↔}BS_{j}\right)$

Let us analyze the protocol to show that UAAPachieves the mentioned goals:

From I1, we have

(26) $$BS_{j}≺{\left\{n_{0},\left(U_{i}\overset{n_{S}^{i}}{↔}BS_{j}\right)\right\}}_{H(X_{S})}$$

The (26), A4 and message meaning rule infers that

(27) $$BS_{j}⊧U_{i}|∼\left\{n_{0},\left(U_{i}\overset{n_{S}^{i}}{↔}BS_{j}\right)\right\}$$

The A1 and freshness conjuncatenation comprehend that

(28) $$BS_{j}⊧\#\left\{n_{0},\left(U_{i}\overset{n_{S}^{i}}{↔}BS_{j}\right)\right\}$$

The (27), (28) and nonce verification rule deduces that

(29) $$BS_{j}⊧\left\{U_{i}⊧n_{S}^{i},n_{0},\left(U_{i}\overset{n_{S}^{i}}{↔}BS_{j}\right)\right\}$$

The (29) and believe rule infers that

(30) $$BS_{j}⊧U_{i}⊧\left(U_{i}\overset{n_{S}^{i}}{↔}BS_{j}\right)$$

From A1, (30) and jurisdiction rule, it concludes

(31) $$BS_{j}⊧\left(U_{i}\overset{n_{S}^{i}}{↔}BS_{j}\right)$$

This belief confirms that $BS_{j}$ has received a message from a legitimate $N_{i}$.

From M2, we have

(32) $$U_{i}≺{\left\{n_{1},,T_{R},U_{i}\overset{K_{S}^{i}}{↔}S_{j},\#\left(U_{i}\overset{K_{S}^{i}}{↔}S_{j}\right),U_{i}\overset{n_{S}^{i}}{↔}BS_{j}\right\}}_{H(X_{S})}$$

The (32), A3 and message meaning rule comprehends that

(33) $$U_{i}⊧BS_{j}|∼\left\{n_{1},,T_{R},U_{i}\overset{K_{S}^{i}}{↔}S_{j},\#\left(U_{i}\overset{K_{S}^{i}}{↔}S_{j}\right)\right\}$$

The (33), A2 and freshness conjuncatenation rule infers that

(34) $$U_{i}⊧\#\left\{n_{1},,T_{R},U_{i}\overset{K_{S}^{i}}{↔}S_{j},\#\left(U_{i}\overset{K_{S}^{i}}{↔}S_{j}\right)\right\}$$

The (33), (34) and nonce verification rule deduce that

(35) $$U_{i}⊧BS_{j}⊧\left\{n_{1},,T_{R},U_{i}\overset{K_{S}^{i}}{↔}S_{j},\#\left(U_{i}\overset{K_{S}^{i}}{↔}S_{j}\right)\right\}$$

The (35) and believe rule infers that (36 Goal-2)

(36) $$U_{i}⊧BS_{j}⊧\left\{U_{i}\overset{K_{S}^{i}}{↔}S_{j},\right\}$$

The (35), (36) and jurisdiction rule concludes that (37 Goal-1)

(37) $$U_{i}⊧\left\{U_{i}\overset{K_{S}^{i}}{↔}S_{j}\right\}$$

From I3, we have

(38) $$BS_{j}≺n_{1}$$

The (36), (37), (38) and meaning rule comprehends that (39 Goal-3)

(39) $$BS_{j}⊧U_{i}⊧\left\{U_{i}\overset{K_{S}^{i}}{↔}S_{j},\right\}$$

### 5.3. Verifying Protocol Using Scyther Tool

The previous section proved that according to the BAN logic the SMSN is a secure authentication scheme. The BAN logic provided a foundation for the formal analysis of security protocols, but few attacks can slip through the BAN logic [32]. For further proof of the strength of the SMSN protocol suite, we implemented the SMSN and [11,12,13,14,15,16] schemes in the automated security protocol analysis tool, Scyther [17,18]. Our proposed scheme provides a strong defense against known attacks in the presence of an intruder (Z) which is capable of regulating the communication channel, redirecting, spoofing, replaying or blocking the messages. It has initially known information, e.g., IDs and public keys of all users, and any intercepted message is additional information to the current information set (S), i.e., S∪{m}. It can generate a fresh message from known data, e.g., S⊢m, and can run multiple instances of the protocol. The Scyther tool verifies the protocol claims and checks the possibility of attacks against the protocol. The claims are the event that describes the design and security properties of the authentication protocol. We consider four claims as defined below; for a detailed description of protocol claims, please refer to [19,20].

In Scyther the protocol is modeled as an exchange of messages among different participating ’roles’; for instance, in sensor node authentication, the sensor node is in the role of initiator, the sink is in the role of responder and the base station is in the role of a server. The Scyther tool integrates the authentication properties into the protocol specification as a claim event. We tested our protocol [11,12,13,14,15,16] employing claims, as mentioned earlier, with the parameter settings given in Table 1.

The results are shown in Table 2. It is clear that in the presence of an intruder (as defined above), our protocol qualifies all the protocol claims and no attacks were found. Hence, for a large number of systems and scenarios, our protocol guarantees safety against a large number of known attacks, such as impersonating, man-in-middle and replay attacks, etc. In contrast, [11,12,13,14,15,16] are susceptible to several attacks and failed to fulfill the authentication claims. Moreover, in protocol schemes [11,12,13,14,15,16], there is a lack of sender-receiver binding verification, and an intruder can exploit this situation to impersonate a sensor node and run multiple instances of the protocol to launch multiplicity and man-in-middle attacks.

## 6. Performance Analysis

Although the SMSN authentication protocol suite covers authentication procedures for both sensor and user nodes, for the sake of simplicity, we compare the efficiency of the SMSN with user authentication protocols. We compared the efficiency of the SMSN user authentication protocols considering computational cost, message complexity and time synchronization requirements to that of [11,12,13,14,15,16]. Unlike in [11,12,13,14,15,16], the SMSN allows the user to be authenticated with both sensor and sink nodes.

The total computation cost is estimated as the sum of the total number of *E* = encryptions/decryptions, *M* = Multiplications, *H* = hash, *X* = XOR, and *T* = Time Synchronization operations. Moreover, we assume that cryptographic hash and symmetric encryption/decryption operations have computational complexity similar to O(m), where m is the size of the message. All the schemes in [11,12,13,14,15,16] considered that the registration process took place via a secure channel. We assume that in all schemes the user node $U_{i}$ and Gateway $GW_{j}$ exchange the registration information using a secure encrypted channel. Furthermore, one encrypted unicast message requires two *E* operations, one for encryption and one for decryption. Similarly, a broadcast message to *N* recipient adds the (N+1)E operations in the total computational complexity.

From Table 3 we can see that computational complexity of SMSN authentication protocols in the registration phase is slightly more expensive compared to Kumar et al. [13] and Farash et al. [15]; however; in the authentication phase, the SMSN authentication protocols completely outperform Kumar et al. [13] and Farash et al. [15]. H. Tseng [11] and Yoo et al. [12] are the most computationally expensive schemes during the registration phase, but in the authentication phase, H. Tseng [11] is the most efficient scheme followed by the SMSN. However, regarding sensor node computational efficiency, in our scheme, the overall workload of a sensor node is very low. Moreover, unlike the [12,13,14,21] schemes, the SMSN does not require time synchronization between the Gateway and user node.

**Figure 13:**
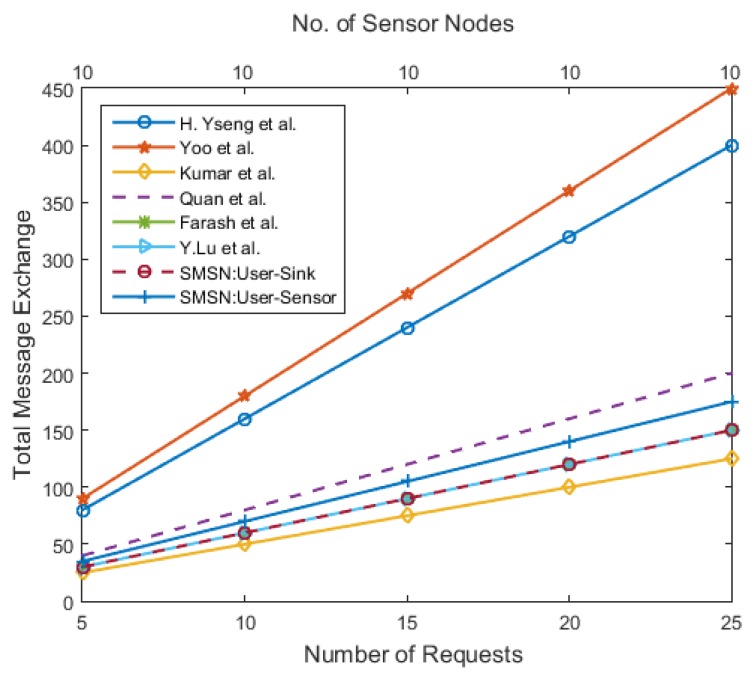
The total number of message exchanged between constant number of sensor nodes with different number of new users’ requests.

**Figure 14:**
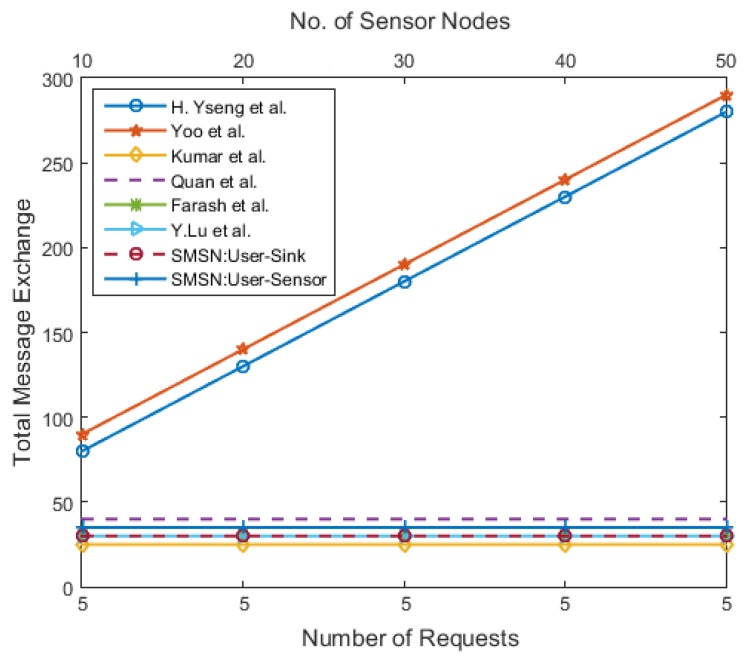
The total number of message exchanged for constant number of new users request in different network size.

The communication complexity is calculated as the sum of the total unicast and broadcast message exchange. Figure 13 shows the overall communication complexity in a WSN when the number of sensor nodes is fixed in the network, and the number of new users’ requests is constantly increasing. The message complexity of [11,12] increases multiplicatively by increasing the number of nodes; conversely, it grows slowly in the case of the SMSN, [13,14,15]. Figure 14 shows the overall communication complexity for various network sizes with the same number of user requests. This metric is only useful for a WSN with mobile sensor and user nodes. The message complexity of [11,12] increases rapidly with an increase in the number of new users’ requests; conversely, it remains constant with the SMSN, [13,14,15]. This suggests that the schemes proposed in [11,12] are not suitable for highly dynamic mobile WSNs where the frequency of leaving and joining the network is high.

**Figure 15:**
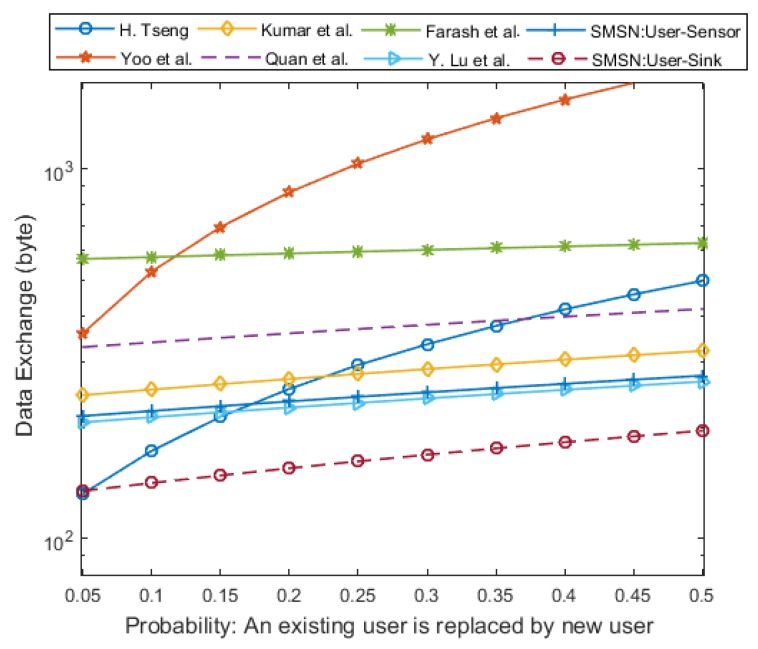
The amount of data exchanged for less to highly dynamic sensor network.

However, a more interesting comparison in terms of communication efficiency is the comparison based on the amount of data exchanged during the protocol run. The numerical results are taken for a dynamic and mobile sensor network consisting of 100 nodes. The probability that a new user may join the network and an existing user may leave the system defines how frequently the users join and leave the network. The average communication cost per user is calculated for the dynamic probability of 0.05 to 0.5, and the results are shown in Figure 15.

SMSN user-sink authentication outperforms all other schemes from less dynamic to highly dynamic networks. However, in a less dynamic network with a dynamic probability of less than 0.05, the SMSN user-sensor authentication is slightly more expensive than the scheme of H. Tseng [11]. Even though in a less dynamic system the SMSN user-sensor authentication is slightly more expensive than [11], the performance gap decreases, and for highly mobile and dynamic networks, SMSN performs better than the scheme of H. Tseng [11].

## 7. Conclusions

Due to the recent growth in WSN technologies, we have observed an enormous paradigm shift in sensor network applications. The authentication and security goals of a sensor network have become more crucial and challenging. Most of the user and sensor node authentication schemes for WSNs have been developed without taking into account the requirements of integrating WSNs with emerging technologies such as IoT. We developed an SMSN scheme considering the requirements of mobile and dynamic WSN applications as discussed in Section-III. We noted that the user authentication schemes designed for sensor networks [11,12,13,14,15,16] do not meet the authentication properties; for example, the execution of these schemes in the Scyther tool revealed that the participating entities failed to achieve wider objectives (defined as protocol claims in Section 5-C) of the authentication protocol. Finally, we compared the efficiency of the SMSN user authentication protocols with the schemes in [11,12,13,14,15,16]. We observed that concerning the computational cost, our scheme is slightly more expensive compared to [13,15] during the registration phase but the SMSN totally outperforms both in the authentication phase. Regarding message complexity our proposed scheme totally outperforms [11,12,13,14,15]; however, the performance of the scheme of Y. Lu et al. [16] is close to the SMSN. Finally, unlike the schemes in [11,12,13,14,15,16], the SMSN does not require time synchronization between the Gateway (base station) and the user node. The main focus of this work was to discuss and provide solutions for the emerging challenge that has emerged from the integration of the WSN in IoT applications. To prove the usability of the proposed scheme, we made a comprehensive security and performance analysis and simulated the proposed idea in an automated protocol verifier tool, the Scyther. However, for future work, it will be interesting to investigate the usability of the SMSN by implementing it on an application specific testbed. Moreover, in the near future it will be possible to incorporate the basic Internet functionality in the sensor node. We believe it will further enhance the application scenarios for the SMSN; for instance a user device will be able to collect real-time sensor data remotely via the Internet. Moreover, we are further investigating the usage of SMSN for the promising future internet architecture, known as Name-Data-Networking (NDN), which is extensively studied in the literature [54,55,56,57,58,59]. In NDN the contents verification is achieved by the use of asymmetric cryptography. We argue that in future especially in IoT application scenarios the devices will be resource constraint devices; for instance, the sensor network is going to be the part of IoT. For resource constraint devices the asymmetric cryptography is computationally expensive. We believe that SMSN protocol suit with some modifications can be a suitable candidate for NDN internet architecture.

## Figures and Tables

**Table 1:** Scyther tool parameter settings.

Parameter	Settings
Number of Runs	1∼3
Matching Type	Find all Type Flaws
Search pruning	Find All Attacks
Number of pattern per claim	10

**Table 2:** Comparison of authentication properties.

Claims	H. Tseng [11]			Yoo et al. [12]			Kumar et al. [13]			Quan et al. [14]			Farash et al. [15]			Y. Lu et al. [16]			SMSN		
	$U_{i}$	$N_{i}$	GW	$U_{i}$	$N_{i}$	GW	$U_{i}$	$N_{i}$	GW	$U_{i}$	$N_{i}$	GW	$U_{i}$	$N_{i}$	GW	$U_{i}$	$N_{i}$	GW	$U_{i}$	$N_{i}$	GW
Aliveness	N	N	N	N	N	N	Y	N	N	Y	N	N	Y	Y	N	N	N	Y	Y	Y	Y
Weak Agreement	N	N	N	N	N	N	Y	N	N	Y	N	N	Y	Y	N	N	N	Y	O	Y	Y
Non-injective Agreement	N	N	N	N	N	N	O	N	N	Y	N	N	Y	Y	N	N	N	Y	O	Y	Y
Non-injective Synch.	N	N	N	N	N	N	O	N	N	Y	N	N	Y	Y	N	N	N	Y	O	Y	Y

N = Authentication claim is not fulfilled; Y = Authentication claim is fulfilled; O = Authentication claim is fulfilled but falsified for protocol instances >3.

**Table 3:** Performance comparison of SMSN with well-known user authentication protocols.

Schemes	Phase	Comp. Complexity	Comm. Complexity	Comm. Cost in Bytes	Time Synch.
H. Tseng [11]	Registration	(4+N)E+1H	2UC+1BC	(2N+3)int	-
	Login-Authentication	6H+1X	4UC	2CK+7int	1T
Yoo et al. [12]	Registration	(4+N)E+5H+2X	2UC+1BC	(5+N)CK+1int	-
	Login-Authentication	19H+2X	6UC	5CK+8int	1T
Kumar et al. [13]	Registration	4H+3X	2UC	5CK+3int	-
	Login-Authentication	14H+2X	3UC	6CK+11int	2T
Quan et al. [14]	Registration	12E+9H+1X+7M	4UC	4CK+9int+4f	4T
	Login-Authentication	18H+2X	4UC	8CK+16int	4T
Farash et al. [15]	Registration	4E+6H+2X	2UC	4CK+1int	2T
	Login-Authentication	30H+16X	4UC	17CK+5int	4T
Y. Lu et al. [16]	Registration	4E+10H+2X	2UC	4CK+1int	-
	Login-Authentication	8E+17H+15X	4UC	4CK+18int	3T
SMSN (User-Sink)	Registration	8E+5H	3UC	3CK+10int	-
	Login-Authentication	8E+2H	3UC	3CK+8int	Optional
SMSN (User-Sensor)	Registration	8E+5H	3UC	3CK+10int	-
	Login-Authentication	9E+2H	4UC	5CK+12int	Optional

*Computational Complexity:* *E* = encryptions/decryptions, Ex = modular exponentiations, *M* = multiplications, *H* = hash operation, *X* = XOR operation , *N* = Total number of nodes , *T* = Time Synchronization operation; *Communication Complexity and Cost:* BC = Broadcast Message, UC = Unicast Message, CK = 32 bytes (Represents Size of Cryptographic KeyHash and Signature), Int = 4 bytes ( Represents Size of nonce, Node Ids and Integers), *f* = 8 bytes (Represents Size of the floating point real number.

## Abbreviations

The following abbreviations are used in this manuscript:

WSN	Wireless Sensor Network
IoT	Internet of Things
SMSN-Protocol	Secure Mobile Sensor Network Protocol
BAN-logic	Burrows Abadi Needham logic
SAAP	Sensor Activation and Authentication Protocol
SRP1	Sensor Re-Authentication Protocol-1
SRP2	Sensor Re-Authentication Protocol-2
UAAP	User Activation and Authentication Protocol
USiAP	User-Sink Authentication Protocol
USeAP	User- Sensor Authentication Protocol
